# Preoperative Prediction of Perineural Invasion and Prognosis in Gastric Cancer Based on Machine Learning through a Radiomics–Clinicopathological Nomogram

**DOI:** 10.3390/cancers16030614

**Published:** 2024-01-31

**Authors:** Heng Jia, Ruzhi Li, Yawei Liu, Tian Zhan, Yuan Li, Jianping Zhang

**Affiliations:** 1 Department of General Surgery, The Second Affiliated Hospital of Nanjing Medical University, Nanjing 210011, China; lbhengjia@163.com (H.J.); 15851863223@163.com (T.Z.); 2 Department of Endoscopic Center, The Fourth Affiliated Hospital of Nanjing Medical University, Nanjing 210031, China; zhizhi1012@njmu.edu.cn; 3 Department of General Surgery, Nanjing Drum Tower Hospital Clinical College of Nanjing Medical University, Nanjing 210008, China; 18262635889@163.com; 4 Key Laboratory of Modern Toxicology, Ministry of Education, School of Public Health, Nanjing Medical University, Nanjing 211166, China

**Keywords:** machine learning, radiomics, gastric cancer, perineural invasion

## Abstract

**Simple Summary:**

Gastric cancer remains the world’s fifth most lethal malignancy. Perineural invasion (PNI) is a common growth pattern of gastric cancer. Currently, the diagnosis of PNI relies on postoperative pathology, which is an invasive approach. In this study, we built a radiomics–clinicopathological model based on logistic regression analysis to preoperatively predict PNI. The radiomics–clinicopathological model yielded AUC values of 0.851 (95%CI: 0.769–0.933) in the training set, 0.842 (95%CI: 0.713–0.970) in the testing set and 0.813 (95%CI: 0.672–0.954) in the validation set. This proposed model may help clinicians make clinical decisions and provide personalized treatment to gastric cancer patients. In this research, the value of perineural invasion (PNI) in predicting prognoses for gastric cancer patients was also studied.

**Abstract:**

Purpose: The aim of this study was to construct and validate a nomogram for preoperatively predicting perineural invasion (PNI) in gastric cancer based on machine learning, and to investigate the impact of PNI on the overall survival (OS) of gastric cancer patients. Methods: Data were collected from 162 gastric patients and analyzed retrospectively, and radiomics features were extracted from contrast-enhanced computed tomography (CECT) scans. A group of 42 patients from the Cancer Imaging Archive (TCIA) were selected as the validation set. Univariable and multivariable analyses were used to analyze the risk factors for PNI. The *t*-test, Max-Relevance and Min-Redundancy (mRMR) and the least absolute shrinkage and selection operator (LASSO) were used to select radiomics features. Radscores were calculated and logistic regression was applied to construct predictive models. A nomogram was developed by combining clinicopathological risk factors and the radscore. The area under the curve (AUC) values of receiver operating characteristic (ROC) curves, calibration curves and clinical decision curves were employed to evaluate the performance of the models. Kaplan–Meier analysis was used to study the impact of PNI on OS. Results: The univariable and multivariable analyses showed that the T stage, N stage and radscore were independent risk factors for PNI (*p* < 0.05). A nomogram based on the T stage, N stage and radscore was developed. The AUC of the combined model yielded 0.851 in the training set, 0.842 in the testing set and 0.813 in the validation set. The Kaplan–Meier analysis showed a statistically significant difference in OS between the PNI group and the non-PNI group (*p* < 0.05). Conclusions: A machine learning-based radiomics–clinicopathological model could effectively predict PNI in gastric cancer preoperatively through a non-invasive approach, and gastric cancer patients with PNI had relatively poor prognoses.

## 1. Introduction

Gastric cancer is one of the most lethal cancers, ranking fifth among all cancers in the world [1]. The current standard treatment for gastric cancer is radical gastrectomy, supplemented by radiotherapy and chemotherapy. However, despite this standard treatment, the overall survival (OS) of gastric cancer patients remains low [2]. Although significant progress has been made in chemotherapy and radiotherapy in recent years, radical gastrectomy remains the most effective therapy. Nevertheless, a large number of gastric cancer patients still experience recurrence and metastasis after radical gastrectomy [3]. Therefore, it is crucial to identify new prognostic factors that contribute to the poor survival of gastric cancer patients, enabling the development of customized treatment plans to enhance patients’ OS.

The TNM staging system is currently recognized as a robust prognostic indicator for gastric cancer [4]. With the continuous study of the clinicopathology and other aspects of gastric cancer patients, some pathological factors and molecular indicators have been confirmed to be related to the prognoses of gastric cancer patients [5,6]. Previous studies suggested that in addition to the TNM stage, perineural invasion (PNI) is an independent factor that affects the prognosis of patients with gastric cancer [7]. PNI is a tumor growth pattern that can lead to poor prognoses such as tumor metastasis and peritoneal recurrence [8]. In gastric cancer, PNI is primarily defined as the circumstance where at least 33% of a nerve is surrounded by tumor cells [9]. The incidence of PNI in gastric cancer varies from 6.9% to 75.6% [10]. Studies have shown that patients with PNI have shorter survival times compared to those without PNI [11]. Consequently, treatments such as surgery or neoadjuvant chemoradiotherapy may be affected if PNI occurs. Currently, the assessment of PNI in gastric cancer relies on post-operative pathology, which is an invasive approach. Therefore, it would be highly significant to preoperatively predict PNI in gastric cancer through a non-invasive approach.

Radiomics has played a crucial role in the diagnosis and treatment of diseases in recent years [12,13]. In gastrointestinal diseases, radiomics is mainly used for tumor staging, differential diagnosis and prognosis analysis [14,15]. Previous studies have explored non-invasive methods for preoperatively assessing PNI in gastric cancer. Zheng et al. investigated the efficacy of radiomics and clinical models based on machine learning for preoperatively predicting PNI in gastric cancer. They found that their approach performed well when identifying PNI before surgery [16]. However, their study did not include enough clinical and pathological factors, and there was no analysis of the impact of PNI on prognosis. Here, we explored the role of a combined radiomics–clinicopathological model in predicting PNI in gastric cancer using machine learning, and analyzed the impact of PNI on prognosis.

## 2. Materials and Methods

### 2.1. Patients

**Figure 1:**
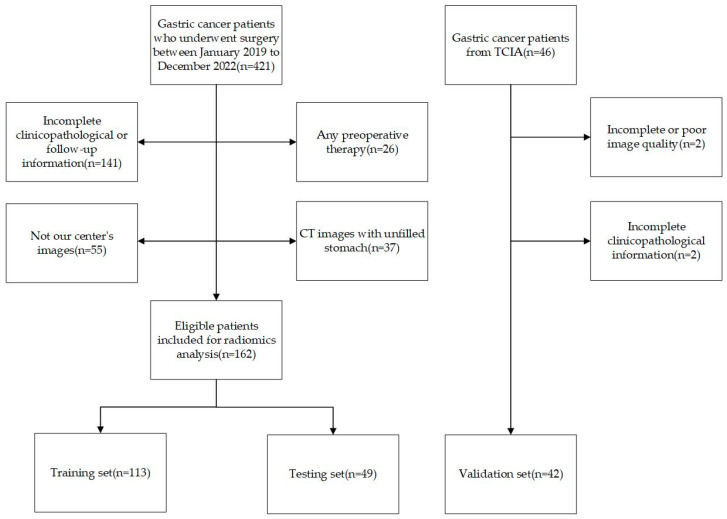
Flow diagram of inclusion and exclusion criteria.

This study analyzed clinicopathological and contrast-enhanced computed tomography (CECT) data from 162 patients treated at the Second Affiliated Hospital of Nanjing Medical University from January 2019 to December 2022. The inclusion criteria were as follows: (1) CECT was performed less than 2 weeks before surgery and (2) the patients had a pathological diagnosis of gastric cancer. The exclusion criteria were as follows: (1) patients with unfilled stomachs; (2) patients with poor CECT quality; (3) patients with combined severe primary disease; and (4) patients with incomplete clinical data or follow-up information. The workflow of the inclusion and exclusion is shown in Figure 1. Our study was approved by the ethics committee of the Second Affiliated Hospital of Nanjing Medical University (2023-KY-162-01), and the patients’ or their family members’ informed consent was acquired. Our study adhered to the Image Biomarker Standardization Initiative (IBSI) guidelines and the Declaration of Helsinki [17].

Patients’ baseline information was collected, including age, gender, tumor location, differentiation type, Lauren type, lymphovascular invasion (LVI) status, smoking status, drinking status, carcinoembryonic antigen (CEA) levels, cancer antigen 125 (CA125) levels, cancer antigen 199 (CA199) levels and human epidermal growth factor receptor 2 (HER-2) levels. Pathological diagnoses were confirmed by two pathologists, and when there was a dispute between the two pathologists, the diagnosis was confirmed by a third pathologist. These patients were randomly divided into a training set and a testing set with a ratio of 7:3, and 42 patients from the Cancer Imaging Archive (TCIA) with complete pathological data were also enrolled as a validation set.

The follow-up information of the 162 patients was also collected via telephone or from outpatient records. The endpoint event was the OS time, which was defined as the time from the day of the surgery to the day of death due to any cause, or 1 October 2023.

### 2.2. CT Image Acquisition

We included the arterial phase in this study because the arterial phase of CECT has better diagnostic performance [18]. All of the patients signed informed consent forms before enhanced CT examinations were carried out. The patients were required to fast for at least 6 h before the CT examination, and drank 1000 mL of water to keep their stomachs dilated. All of the patients underwent 64-slice dual source CT. Patients received 1.5 mL/kg of an iodinated contrast agent (Ioversol Injection 320 mg I/mL, Jiangsu Hengrui Pharmaceuticals Co. Ltd., Lianyungang, China) at a flow rate of 3.0 mL/sec using an automatic syringe pump. The arterial-phase imaging and venous-phase imaging followed a 30-s and a 60-s delay after the intravenous injection, respectively. The scan parameters were as follows: tube voltage, 120 kV; tube current, 150–300 mA; field of view, 30–50 cm; matrix, 512 × 512; rotation time, 0.5 s; and pitch, 1.0. The images were reconstructed with section thicknesses of 2 mm.

### 2.3. Regions of Interest and Extraction of Radiomics Features

We obtained Digital Imaging and Communications in Medicine (DICOM)-format images from the Picture Archiving and Communication System (PACS), and two experienced radiologists manually drew regions of interest (ROIs), slice by slice, using ITK-SNAP software (version 3.8.0) [19]. One radiologist manually drew ROIs for all of the patients, while the other draw ROIs for 30 randomly selected patients. The Python package PyRadiomics (version 2.1.2) was used to extract radiomics features from the ROIs [20]. All private patient information was removed. A Laplace of Gaussian filter with sigma values of 3 mm, 4 mm and 5 mm was used to reconstruct the images. The Pingouin package was utilized to calculate the intraclass coefficients (ICCs), and features with ICCs > 0.75 were considered effective [21].

### 2.4. Feature Selection and Calculation of Radscores

A total of 1595 features were extracted from ROIs delineated using ITK-SNAP software. The Synthetic Minority Over-Sampling Technique (SMOTE) was used to manage the imbalanced data in the training set. A Z-score transformation was used to normalize the features to the same level. First, a *t*-test was conducted to identify features that distinguish the PNI group from the non-PNI group. Second, the Max-Relevance and Min-Redundancy (mRMR) approach was utilized to filter irrelevant features, and the top 15 features were selected for further consideration. Finally, the least absolute shrinkage and selection operator (LASSO) algorithm was used to select features, and a 10-fold cross-validation was performed to obtain the optimal lambda. The features’ radscores were calculated based on their coefficients and their values via the following: Radscore = $\sum_{i=0}^{n}C_{i}\times X_{i}$ + b, where *C_i_* is the coefficient of the *i*-th selected feature, *X_i_* is the value of the selected feature and b is the intercept.

### 2.5. Model Construction and Evaluation

**Figure 2:**
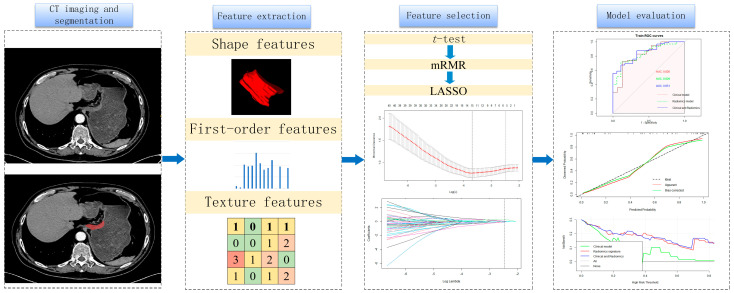
Workflow of perineural invasion prediction. A total of 1595 features were extracted from arterial phases of each patient. The *t*-test, Max-Relevance and Min-Redundancy (mRMR) and the least absolute shrinkage and selection operator (LASSO) were used to select features. Receiver operating characteristics (ROCs), calibration curves and clinical decision curves were used to evaluate the models.

Univariable and multivariable analyses were conducted, combining the radscore and clinicopathological variables. A nomogram was developed to predict PNI by combining clinicopathological and radiomics features. The performance of the model was evaluated based on area under receiver operating characteristics (ROC) curves (AUCs). The calibration curve measured the consistency between the predicted and actual probabilities of PNI. Clinical decision curves were used to analyze and evaluate the clinical practicality of the model. Overall, calibration curves and clinical decision curves were used to evaluate the model’s efficacy. The workflow of our study is shown in Figure 2.

### 2.6. Statistical Analysis

Statistical analyses were performed using Python (version 3.7.0), R software (version 4.2.3) and SPSS (version 22.0) software. Kaplan–Meier analyses were performed to assess OS. The *t*-test was used to test normally distributed continuous variables, and the Mann–Whitney U test was used to test non-normally distributed continuous variables. The chi-square test and Fisher test were used to test categorical variables. The R package “glmnet” was used to perform a LASSO algorithm analysis. The R package “rms” was used to draw the nomogram and the calibration curves. The R package “pROC” was used to draw ROC curves, and R package “dca.R” was used to analyze clinical decision curves. The R package “shiny” and “DynNom” were utilized to construct the online nomogram.

## 3. Results

### 3.1. Clinical Characteristics

**Figure 3:**
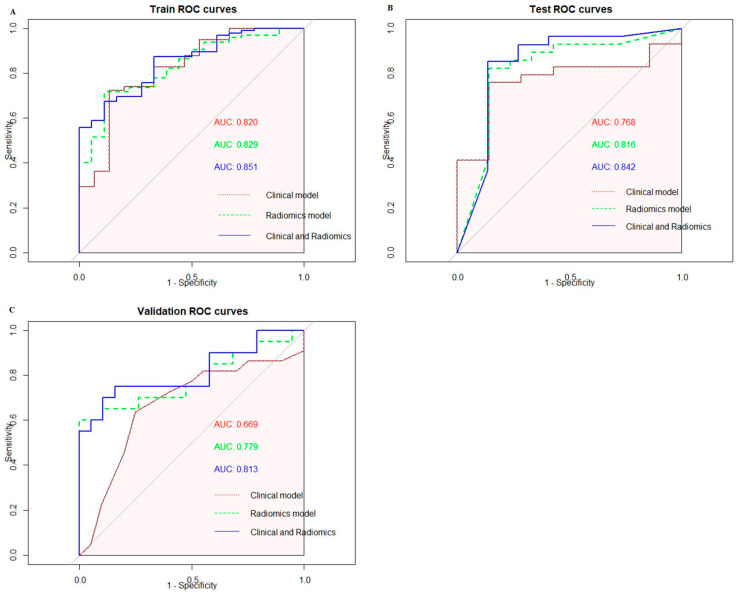
(**A**) ROC curves of three models in the training set. (**B**) ROC curves of three models in the testing set. (**C**) ROC curves of three models in the validation set.

A total of 162 patients were included in this study. The patients were randomly split into a training set and a testing set with a ratio of 7:3, with 113 patients in the training set and 49 patients in the testing set. Among the 113 patients, 95 patients were pathologically diagnosed with PNI and 18 patients were not. In the testing set, 33 patients were diagnosed with PNI and 16 patients were not. The differences in the clinical and pathological factors are shown in Table 1. According to the univariable analysis, the tumors’ T stage (*p* < 0.01), N stage (*p* < 0.01) and LVI (*p* = 0.042) were closely related to PNI. We included the T stage, N stage and LVI to build clinical models based on a logistic regression. Additionally, 42 patients from TCIA were recruited as the validation set. As shown in Figure 3, the AUCs of the clinical models with the logistic regression were 0.820 (95%CI: 0.695–0.944) in the training set and 0.768 (95%CI: 0.596–0.491) in the testing set. The clinical model was validated using the validation set, and the AUC was 0.669 (95%CI: 0.497–0.842).

### 3.2. Radiomics Features Selection and Model Construction

**Figure 4:**
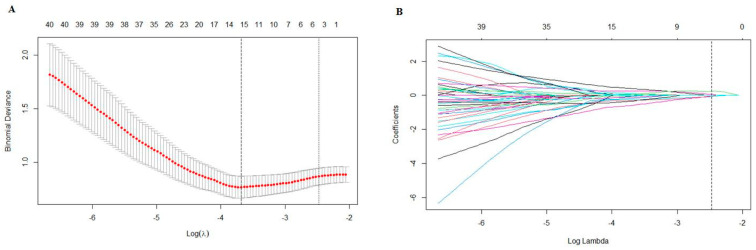
(**A**) A 10-fold cross validation was used to select the optimal “Lambda” value. (**B**) The different colors referred to different features. The best “Lambda” value was used to select features.

A total of 1595 features were extracted using PyRadiomics, including 14 shape features, 306 first-order features and 1275 texture features. The texture features consisted of 408 gray-level co-occurrence matrix (glcm) features, 238 gray-level dependence matrix (gldm) features, 272 gray-level run-length matrix (glrlm) features, 272 gray-level size-zone matrix (glszm) features and 85 neighboring gray-tone difference matrix (ngtdm) features. After performing the *t*-test, 224 features were retained. Subsequently, the Max-Relevance and Min-Redundancy (mRMR) approach was utilized to select features, and the top fifteen features were chosen for the least absolute shrinkage and selection operator (LASSO) analysis. A 10-fold cross validation was used to select the optimal “Lambda” value, and the best “Lambda” value was used to select features (Figure 4). After three rounds of feature selection, five features (wavelet.HLL_glcm_InverseVariance, original_firstorder_90Percentile, wavelet.HHH_firstorder_Minimum, wavelet.LLL_firstorder_Median and gradient_gldm_DependenceNonUniformityNormalized) were ultimately used to build the radiomics models. The coefficients of the selected features are shown in Table 2. The radiomics model, based on the five features, exhibited AUC values of 0.829 (95%CI: 0.738–0.921) in the training set, 0.816 (95%CI: 0.683–0.950) in the testing set and 0.779 (95%CI: 0.625–0.933) in the validation set (Figure 3).

### 3.3. Construction and Evaluation of Combined Radiomics–Clinicopathological Model

According to the univariable analysis, the T stage, N stage, LVI and radscore were associated with PNI (*p* < 0.05). According to the multivariable analysis, the T stage, N stage and radscore were independent risk factors for PNI (*p* < 0.05), and the OR values of the T stage, N stage and radscore were 8.013 (95%CI: 2.604–24.660), 2.882 (95%CI: 1.266–6.564) and 3.040 (95%CI: 1.250–7.397), respectively (Table 3). The combined radiomics–clinicopathological model based on the T stage, N stage and radscore showed robust efficacy in predicting PNI. The combined radiomics–clinicopathological model was superior in predicting PNI compared to both the clinical model and the radiomics model; the AUC values of the radiomics–clinicopathological model were 0.851 (95%CI: 0.769–0.933) and 0.842 (95%CI: 0.713–0.970) in the training set and testing set, respectively. The AUC value of the radiomics–clinicopathological model in the validation set was 0.813 (95%CI: 0.672–0.954). The ROC curves of the training set, testing set and validation set are shown in Figure 3. The detailed model performance is shown in Table 4.

**Figure 5:**
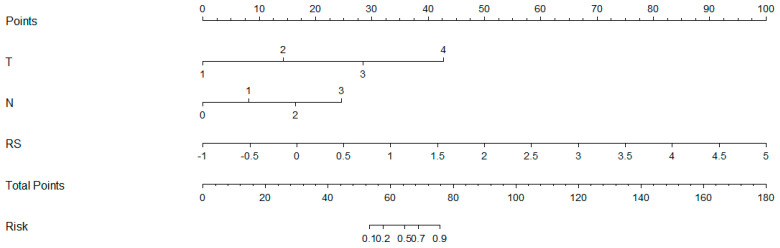
The nomogram of the radiomics-clinicopathological model.

**Figure 6:**
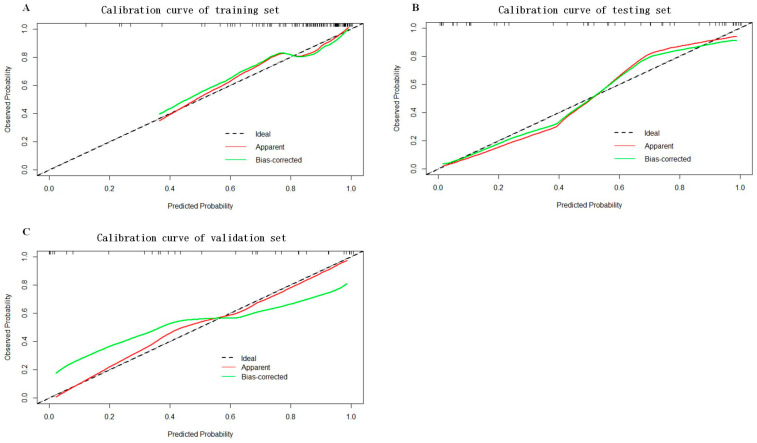
The calibration curves of the training set (**A**), testing set (**B**) and validation set (**C**).

**Figure 7:**
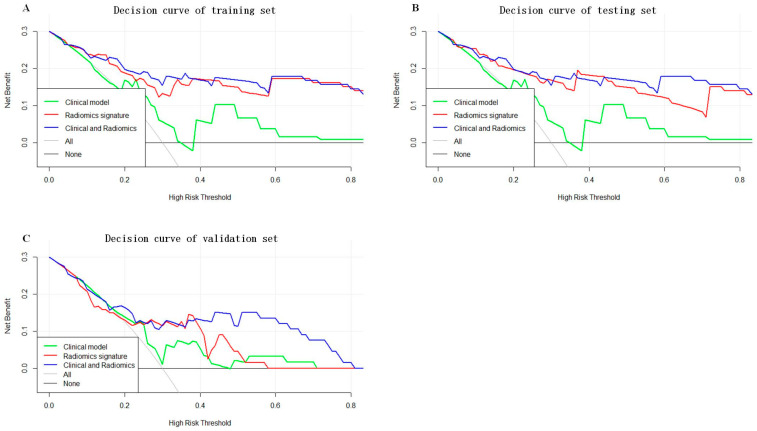
The decision curves of the training set (**A**), testing set (**B**) and validation set (**C**).

As shown in Figure 5, a nomogram of the radiomics–clinicopathological model was developed. In order to improve the generalizability and clinical application value of the model, we designed an online nomogram, and the website is as follows: https://lbhengjia.shinyapps.io/PNI_Predict/ (accessed on 25 January 2024). The calibration curve showed a good balance between the observed and predicted probability scores. The decision curves showed that the combined model provided a greater benefit than the clinical and radiomics models (Figure 6 and Figure 7).

### 3.4. Survival Analysis

**Figure 8:**
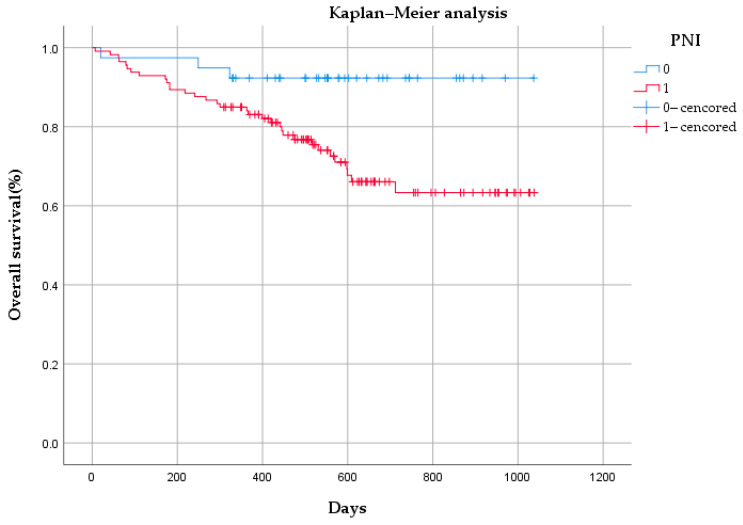
Kaplan–Meier analysis of overall survival based on perineural invasion (PNI) status among 162 patients.

The median OS time of the 162 patients was 542 days (range: 7–1038 days). Of the 162 patients, 34 patients had no PNI, and 3 among them died. In total, 128 patients had PNI, and there were 33 deaths in this group. As shown in Figure 8, there was a statistically significant difference in OS between the PNI group and the non-PNI group (*p* < 0.05).

## 4. Discussion

Early onset gastric cancer often lacks specific symptoms, and patients with gastric cancer often present in advanced stages when seeking medical attention. The overall 5-year survival rate of patients with advanced gastric cancer is relatively low, and the overall prognosis is poor. The degree of tumor infiltration, lymph node metastasis and tumor size are the most critical factors affecting the postoperative survival rate of gastric cancer, but many related studies have shown that the prognoses of gastric cancer patients are also related to LVI and PNI [22,23]. Currently, relevant studies have shown that LVI is one of the independent factors affecting the prognoses of gastric cancer patients, while there is relatively little research on PNI [24]. PNI is one of the biological features of gastric cancer. Patients with PNI always have worse outcomes compared to patients without PNI [25]. Whether PNI is an independent risk factor affecting the prognoses of gastric cancer patients is still under debate. At the same time, the current criteria of PNI mainly rely on postoperative pathology, which is an invasive method with a time lag that has little significance in the selection of a treatment mode. In this research, we developed a radiomics model to preoperatively predict the PNI of gastric cancer, which has more guiding significance for the treatment mode and surgical approach. Therefore, this study attempted to use artificial intelligence methods to non-invasively predict the PNI of gastric cancer before surgery, and to determine its value in predicting the prognoses of gastric cancer patients.

In terms of the specific mechanism of the PNI of gastric cancer, Kai Yin et al. confirmed that the axon guidance molecule promoted gastric cancer cell navigation along peripheral neuritis [26]. Jia X et al. analyzed the expression of PNI gene signatures in gastric cancer via a meta-analysis of gene expression profiling, and found that genes regulating cell adhesion molecules were upregulated and associated with poor survival in gastric cancer [27]. Currently, there is relatively little research on the mechanism of the PNI of gastric cancer, and more basic experiments are still needed to elucidate this mechanism. At the same time, there are a few studies that have focused on the preoperative assessment of PNI in patients with gastric cancer via radiomics. Yardımcı analyzed texture features of gastric cancer based on multiple machine learning models. However, this study had a small sample size and did not analyze clinical data, and the models had poor efficacy [28]. In this study, we included a cohort of 204 patients and enough clinicopathological risk factors to obtain more convincing results.

In our study, we found that advanced T stage and N stage classifications were closely associated with the PNI of gastric cancer, which was consistent with the results of a previous study [29]. There were no other covariates associated with PNI, and studies with large sample sizes need to be further developed in the future. The AUC of the clinical model (including the T stage and N stage) reached 0.820 in the training set and 0.768 in the testing set, indicating that the above clinical factors provided a high level of accuracy when predicting PNI. Adding radiomics features to the clinical model could help improve performance, as the combined model had AUC values of 0.851 and 0.842 in the training set and testing set, respectively. The combined model was also validated using an external validation dataset, achieving an AUC value of 0.813. The relatively inferior performance of the combined model on the validation set compared to the training set and testing set may be attributed to the smaller sample size and the variable image quality across datasets. At the same time, our study also found that gastric cancer patients with PNI had poorer prognoses than those without PNI, which was in line with a previous study [30].

In recent years, the integration of radiomics and machine learning has shown great potential to advance our understanding of various gastric cancer conditions, especially PNI. In our research, we focused on exploring the mechanisms of PNI using radiomics and machine learning methods. By analyzing various shape and texture features in radiomics images, we could analyze the PNI of tumors from both macro and micro perspectives. Among the radiomics features we included, there were three wavelet features. These wavelet features can provide information about the internal vascular structure, tissue density and microscopic environments of tumors, and can thereby help us better understand the mechanisms of PNI and improve the model’s predictive ability [31]. By synthesizing these multi-dimensional data, we can obtain more comprehensive and accurate tumor features and establish more reliable models to predict the PNI of gastric cancer. Furthermore, we plan to prospectively validate the developed model’s effectiveness and evaluate its potential impact on clinical decision making and personalized treatment strategies.

Our study also has many limitations. Firstly, this was a single-center study with relatively small sample sizes; a multicenter study with large samples needs to be conducted. Secondly, our study did not investigate the specific mechanism of PNI through basic experiments. Thirdly, the manual delineation of ROIs presented a certain degree of heterogeneity, and a deep learning model without manual delineation needs to be further developed.

## 5. Conclusions

Gastric cancer patients with PNI have relatively poor prognoses; radiomics–clinicopathological models can effectively predict the PNI of gastric cancer, which could help clinicians in diagnosis and decision making.

## Figures and Tables

**Table 1:** Clinical and pathological characteristics of recruited patients.

		Training Set		*p*	Testing Set		*p*
		PNI (+)	PNI (−)		PNI (+)	PNI (−)	
		N = 95	N = 18		N = 33	N = 16	
Age (mean ± SD ^1^)		66.11 ± 10.85	65.72 ± 8.85	0.194	62.91 ± 11.38	67.8 ± 3.42	0.494
Gender, No. (%)				0.350			0.218
	Male	70 (73.7%)	10 (55.6%)		26 (78.8%)	5 (31.3%)	
	Female	25 (26.3%)	8 (44.4%)		7 (21.2%)	11 (68.7%)	
Size, No. (%)				0.191			0.189
	<5 cm	45 (47.4%)	12 (66.7%)		18 (54.5%)	13 (81.2%)	
	≥5 cm	50 (52.6%)	6 (33.3%)		15 (45.5%)	3 (18.8%)	
Location, No. (%)				0.315			0.205
	Antrum	37 (38.9%)	9 (50%)		7 (21.2%)	4 (25.0%)	
	Body	27 (28.4%)	2 (11.1%)		5 (15.2%)	5 (31.3%)	
	Fundus	31 (32.6%)	7 (38.9%)		21 (63.6%)	7 (43.8%)	
Tissue differentiation, No. (%)				0.119			0.075
	High	51 (53.7%)	5 (27.8%)		18 (54.5%)	9 (56.3%)	
	Middle	28 (29.5%)	9 (50%)		10 (30.3%)	6 (37.5%)	
	Low	16 (16.8%)	4 (22.2%)		5 (15.2%)	1 (6.3%)	
Lauren type, No. (%)				>0.9			0.051
	Intestinal	20 (21.5%)	9 (50.0%)		5 (15.2%)	2 (12.5%)	
	Diffuse	35 (37.6%)	3 (16.7%)		11 (33.3%)	1 (6.3%)	
	Mixed	38 (40.9%)	6 (33.3%)		17 (51.5%)	13 (81.3%)	
T stage, No. (%)				<0.001			0.008
	1	11 (11.6%)	5 (27.8%)		8 (24.2%)	12 (75.0%)	
	2	7 (7.37%)	7 (38.9%)		2 (6.1%)	3 (18.8%)	
	3	43 (45.3%)	2 (11.1%)		13 (39.4%)	1 (6.3%)	
	4	34 (35.8%)	4 (22.2%)		10 (30.3%)	0	
N stage, No. (%)				<0.001			0.003
	0	7 (7.37%)	10 (55.6%)		5 (15.2%)	3 (18.8%)	
	1	24 (25.3%)	1 (5.56%)		3 (9.1%)	12 (75%)	
	2	18 (18.9%)	5 (27.8%)		9 (27.3%)	1 (6.3%)	
	3	46 (48.4%)	2 (11.1%)		16 (48.5%)	0	
LVI ^2^, No. (%)				0.014			0.001
	Yes	58 (61.1%)	8 (44.4%)		25 (75.8%)	12 (75%)	
	No	37 (38.9%)	10 (55.6%)		8 (24.2%)	4 (25%)	
HER-2 ^3^, No. (%)				0.847			0.881
	(0–1+)	54 (74.0%)	11 (61.1%)		19 (57.6%)	5 (31.3%)	
	(++–+++)	19 (26.0%)	7 (38.9%)		14 (42.4%)	11 (68.7%)	
Neutrophils, median (IQR)		4.09 (2.79, 4.69)	3.56 (2.73, 4.27)	0.417	3.63 (3.03, 4.74)	3.19 (2.83, 5.35)	0.706
Lymphocytes, median (IQR)		1.46 (1.14, 1.80)	1.33 (1.19, 1.87)	0.812	1.51 (1.03, 1.96)	1.39 (1.22, 1.82)	1.000
Albumin, median (IQR)		39.80 (36.75, 41.60)	42.00 (35.85, 44.35)	0.460	41.5 (35.9, 45.5)	38.1 (36.45, 40.4)	0.448
CEA ^4^, No. (%)				0.374			0.545
	≤10	90(95.2%)	16(88.9%)		27 (81.9%)	14 (87.5%)	
	>10	5(4.8%)	2(11.1%)		6 (18.2%)	2 (12.5%)	
CA125 ^5^, No. (%)				0.133			0.628
	≤35	83 (87.3%)	16 (88.9%)		19 (57.6%)	10 (62.5%)	
	>35	8 (12.7%)	2 (11.1%)		14 (42.4%)	6 (37.5%)	
CA199 ^6^, No. (%)				0.096			0.545
	≤37.0 U/mL	71 (74.6%)	15 (83.3%)		27 (81.8%)	10 (62.5%)	
	>37.0 U/mL	24 (25.4%)	3 (16.7%)		6 (18.2%)	6 (37.5%)	
Smoking history, No. (%)				0.983			0.245
	Yes	8 (12.7%)	2 (11.1%)		29 (87.9%)	4 (25.0%)	
	No	83 (87.3%)	16 (88.9%)		4 (12.1%)	12 (75.0%)	
Drinking history, No. (%)				0.876			0.262
	Yes	11 (11.6%)	4 (22.2%)		30 (90.9%)	1 (6.3%)	
	No	84 (88.4%)	14 (77.8%)		3 (9.1%)	15 (93.7%)	
Radscore, median (IQR)		1.74 (1.58, 1.99)	1.43 (1.14, 1.60)	<0.001	1.90 (1.69, 2.07)	1.50 (1.33, 1.64)	<0.001

^1^ standard deviation; ^2^ lymphovascular invasion; ^3^ human epidermal growth factor receptor 2; ^4^ carcinoembryonic antigen; ^5^ cancer antigen 125; ^6^ cancer antigen 199.

**Table 2:** The coefficients of the selected features.

Features	Coefficients
wavelet.HLL_glcm_InverseVariance	0.17618597
original_firstorder_90Percentile	0.06464257
wavelet.HHH_firstorder_Minimum	−2.96234129
wavelet.LLL_firstorder_Median	0.84062825
gradient_gldm_DependenceNonUniformityNormalized	1.20886446

**Table 3:** Results of predicted factors and their odds ratio (OR) values in the multivariate analysis.

Predicted Factors	OR ^1^	95%CI ^2^	*p*-Value
Age	1.008	0.961–1.057	0.754
Gender	0.786	0.272–2.270	0.656
Size	1.031	0.846–1.258	0.760
Location	1.086	0.638–1.847	0.762
Tissue differentiation	0.646	0.352–1.185	0.158
Lauren type	1.261	0.693–2.296	0.447
T stage	8.013	2.604–24.660	0.001
N stage	2.882	1.266–6.564	0.012
LVI ^3^	1.344	0.162–11.130	0.784
HER-2 ^4^	0.831	0.302–2.286	0.720
Neutrophils	0.981	0.840–1.146	0.981
Lymphocytes	1.028	0.432–2.448	0.950
Albumin	1.007	0.923–1.100	0.870
CEA ^5^	1.091	0.925–1.288	0.300
CA-125 ^6^	1.046	0.980–1.116	0.181
CA-199 ^7^	1.027	0.990–1.065	0.154
Smoking history	0.432	0.074–2.531	0.352
Drinking history	2.169	0.260–18.120	0.475
Radscore	3.040	1.250–7.397	0.014

^1^ odds ratio; ^2^ confidence interval; ^3^ lymphovascular invasion; ^4^ human epidermal growth factor receptor 2; ^5^ carcinoembryonic antigen; ^6^ cancer antigen 125; ^7^ cancer antigen 199.

**Table 4:** The detailed information for the three models.

	Training Set (n = 113)				Testing Set (n = 49)				Validation Set (n = 42)			
	AUC (95%CI)	ACC	SEN	SPE	AUC (95%CI)	ACC	SEN	SPE	AUC (95%CI)	ACC	SEN	SPE
Model1	0.820 (0.695–0.944)	0.832	0.851	0.400	0.768 (0.596–0.941)	0.837	0.851	0.500	0.669 (0.497–0.842)	0.619	0.600	0.667
Model2	0.829 (0.738–0.921)	0.876	0.879	0.833	0.816 (0.683–0.950)	0.836	0.851	0.772	0.779 (0.625–0.933)	0.718	0.737	0.700
Model3	0.851 (0.769–0.933)	0.929	0.886	0.750	0.842 (0.713–0.970)	0.837	0.851	0.818	0.813 (0.672–0.954)	0.744	0.750	0.737

Model1: clinicopathological model; Model2: radiomics model; Model3: combined model; AUC: area under curve; ACC: accuracy; SEN: sensitivity; SPE: specificity.

## Data Availability

The data supporting this study are available from the corresponding author upon reasonable request.
